# “I Lived Life” - a Memoir by Unaiza Niaz

**Published:** 2020

**Prof. Unaiza Niaz** is an iconic figure in Psychiatry in Pakistan. Her contributions to Women Mental Health in Pakistan and internationally are well acknowledged. She is a role model for young female psychiatrists, as their number grows, especially in the ranks at Pakistan Psychiatric Society.

Dr. Unaiza Niaz has a leadership position in the field of Women’s Mental Health and she is the Founding Member of Sections on Women’s Mental Health (WMH) in World Psychiatric Association (WPA), International Association for Women’s Mental Health (IAWMH), and World Federation of Mental Health (WFMH).

I lived life is a beautiful publication of Dr. Niaz’s Memoirs. It is an elegant, strikingly artistic gift! This sets a precedence of tasteful, artistic presentation and quality printing of autobiographies; which are adorned with rare photographs and meaningful poetry of spiritual mystics and Sufi’s adds a new dimension to author’s vision and spiritual life.

This memoir poignantly, gives glimpses of changing cultural and social values, in the last several decades in Pakistan. Her pride and joy in quoting her ancestors, grandparents and parents is definitely worth a read! The struggles of a single Muslim girl, in early 1970’s, both in England and America, keeping her strong moral and cultural values was no easy deal. Medical Ethics and education have been her forte throughout her career.

Working and training in centers of excellence as the Royal Free Hospital London, Tavistock Center of Human Relations, Hampstead, The Johns Hopkins University Hospital Baltimore, USA and developing a longstanding relationship with her colleagues is indeed rare. Dr. Niaz ‘s outstanding academic and professional achievements in Psychotrauma, women’s mental health and aviation psychiatry, makes her a unique internationally acclaimed woman psychiatrist.(Validated by several awards and high positions in professional organizations WPA, WFMH,ISTSS,& IAWMH ) She has authored more than ten books on different aspects of her profession, more than 120 publications and chapters in several textbooks which is no mean achievement. Lasting parental influence, with strong faith & spiritual strength, noticeably facilitated Dr. Niaz in her life struggles and led her to be a content and humble human being.

Her autobiography has been acclaimed by healthcare professionals and earned good reviews. Given below are comments received from some healthcare professionals and others from different segment of society.

Lt. Col. Inam Ul Haque Masood (Retd) Consultant Psychiatrist says that “There are few people in everyone’s lifespan whose mere vibrancy keeps the very fiber of one’s life a rainbow ride, an ecstasy spell and a desirous feat. One such person has been Prof. Dr. Unaiza Niaz whose very life, as I know it since ages, has been a life lived fantastically indeed. A life that was from and for the wonderland that did sprout from within her and extended the very touch of magic to those who did come in contact with its very fiber. Going through her autobiography brought back all these wonders, of mine, associated with this enchantress of life in a single stroke which have been dimmed by the mist of bygone times and whose rejuvenation through this book was an absolute delight at the very least and I couldn’t resist telling myself that this life of hers has indeed been “A Lived Life”.

The autobiography does shed ample light on every single aspect of her life, a phenomenal experience. With her professional and academic accolades, which have been more than enough to spellbind anyone in a single flash and about whom the book itself is self-depictive, she has been a lady, like none other even in my extended circle. A torch bearer of the family tradition of knowledge, an activist in action who has been grooming it further and an effective medium channelizing it in all its phenomenal ecstasies to the next generation, thus keeping the chain of career and character builders of society intact.

Apart from the professional medals associated with her life she, has several other feathers added to her hat too which have been well deserved and well earned through a lifetime of jubilant fight at various fronts based on her unending chivalry. Honest and truth bound to her very base I’ve seen her raise the flag of dissent even on the very hostile fronts without any hesitation. She has always fought for the mental wellbeing of women and children in a society, where both these strata of society are governed through social and domestic dictatorship and are not considered evolved enough to have issues associated with their mental and/or psychological prestige in any human way.

Hence, with her battles against tyranny and ignorance, with all her winnings against crumbling traditions and blind resistance and with all her glare and aura amidst darkness and gloom she indeed had “A Lived Life” which itself is nothing less than the guiding force of a lighthouse for those following her footsteps”.

Prof. Qudsia Tariq, Chairperson Department of Psychology University of Karachi says that “Flipping through the pages and reading about her struggles and ventures only brought out stronger emotions. I was thoroughly moved and impressed by how well-documented her memoir was, especially how carefully curated all the pictures were. I never realized how much I didn’t know about her. Her story is that of resilience, of love, of grief, of glory, of successes, and of getting back up after a hard knock out by life. The way she’s penned down her words, I felt like I was on this journey with her. I still can’t wrap my head around the fact that she’s safely kept pictures from every stage of her life. It’s truly amazing!

Her autobiography is definitely a visual and emotional treat for anyone who reads her book! I would highly recommend it to anyone who appreciates reading about success stories about people who build themselves from the ground up. Stories that are raw, honest, real and inspiring”.

**Dr. Habiba Hasan** Consultant Pediatrician writes that “I Lived Life is a pictorial autobiography of a talented high achiever – Dr. Unaiza Niaz. She traces her life from its roots in the ancestors both on the maternal and paternal side to her successful life today. This position she has achieved through sheer dedication and hard work, undeterred by the setbacks due to early death of both her parents and siblings. She managed to get training at the top medical institutions both in UK and the US. Upon joining PIA, she did courses in Aviation Psychiatry, thereby becoming the most qualified Aviation Psychiatrist in Pakistan. Going through the book brought back many memories. The book’s get up and printing is excellent for which she deserve to be congratulated “.

**Dr. Fawzia Usmani**, a General Physician in her review of the autobiography says that “Memoirs of Prof. Unaiza Niaz is a beautiful book! It makes quite an elegant, strikingly artistic gift! It is much more than display! Dr. Niaz’s Memoir succinctly identifies the dilemma of growing young women soon after the independence of Pakistan.”

**Ms. Aisha Rahim, a** School Teacher commenting on the autobiography says that “The layout is beautiful, starting from the cover page which is truly impressive, the pages are like an artist’s palette with refreshing pictures of flowers, thought provoking poetry and quotes… The pictures speak volumes and one can vividly visualize her. The book is beautifully written and truly inspiring.

**Mr. Pervez Iqbal** a noted Publisher has commented that “using textured ground on a silky paper for her autobiography with appropriate selection of fonts is commendable. The selection of photographs with crisp captions has made this autobiography an easy read. The beautiful portrait of the author by non-other than Iqbal Mehdi is the best if one is to select the most appealing image in the entire book. The episode with Professor Gerald Russell must be amusing for a reader. Quotes by one of the greatest spiritual masters and poetical intellects “RUMI” has added invaluable depth and helping a reader to understand the background of each reference the author has made. Finally, the selection of Quranic Verses is aptly done to express the gratitude to The Creator of this universe.”

Members of the medical profession in general and those in the field of mental health as well as family physicians, postgraduates and medical students will find it extremely informative and useful. It will help them understand how to live a successful life in today’s Pakistan and the world at large and face the difficult situations with courage and determination with a positive outlook.

**ISBN:** 978-969-2341-0-0-4. Price not given. Private Publication- Not for sale. Those interested can contact on E-mail: drunaiza@gmail.com.

